# Genomic and biochemical analysis of repeatedly observed variants in *DBT* in individuals with maple syrup urine disease of Central American ancestry

**DOI:** 10.1002/ajmg.a.62893

**Published:** 2022-07-07

**Authors:** Charles J. Billington, Kimberly A. Chapman, Eyby Leon, Beatrix W. Meltzer, Seth I. Berger, Matthew Olson, Robert A. Figler, Steve A. Hoang, Cui Wanxing, Brian R. Wamhoff, M. Sol Collado, Kristina Cusmano‐Ozog

**Affiliations:** ^1^ Children's National Rare Disease Institute Washington District of Columbia USA; ^2^ Department of Pediatrics University of Minnesota Minneapolis Minnesota USA; ^3^ Laboratory Medicine, Children's National Hospital Washington District of Columbia USA; ^4^ HemoShear Therapeutics, Inc. Charlottesville Virginia USA; ^5^ Georgetown University Hospital Washington District of Columbia USA; ^6^ Department of Pathology Stanford University Medical Center Stanford California USA

**Keywords:** branched‐chain alpha‐ketoacids, *DBT*, maple syrup urine disease

## Abstract

Maple syrup urine disease (MSUD) is an intoxication‐type inherited metabolic disorder in which hyperleucinemia leads to brain swelling and death without treatment. MSUD is caused by branched‐chain alpha‐ketoacid dehydrogenase deficiency due to biallelic loss of the protein products from the genes *BCKDHA*, *BCKDHB*, or *DBT*, while a distinct but related condition is caused by loss of *DLD*. In this case series, eleven individuals with MSUD caused by two pathogenic variants in *DBT* are presented. All eleven individuals have a deletion of exon 2 (delEx2, NM_001918.3:c.48_171del); six individuals are homozygous and five individuals are compound heterozygous with a novel missense variant (NM_001918.5:c.916 T > C [p.Ser306Pro]) confirmed to be in trans. Western Blot indicates decreased amount of protein product in delEx2;c.916 T > C liver cells and absence of protein product in delEx2 homozygous hepatocytes. Ultrahigh performance liquid chromatography–tandem mass spectrometry demonstrates an accumulation of branched‐chain amino acids and alpha‐ketoacids in explanted hepatocytes. Individuals with these variants have a neonatal‐onset, non‐thiamine‐responsive, classical form of MSUD. Strikingly, the entire cohort is derived from families who immigrated to the Washington, DC, metro area from Honduras or El Salvador suggesting the possibility of a founder effect.

## 1 INTRODUCTION

**FIGURE 1:**
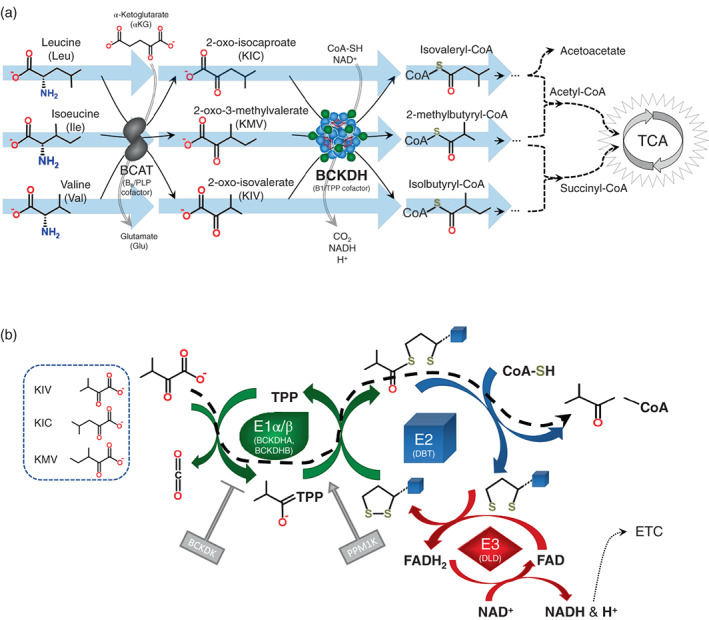
(a) the branched‐chain amino acids, isoleucine, leucine, and valine are metabolized in a multi‐step process into succinyl‐ and acetyl‐CoA as entry points into the energy‐generating tricarboxylic acid (TCA) cycle. The initial step is deamination of the branched‐chain amino acids to alpha‐ketoacids. Subsequently, branched‐chain alpha‐ketoacid dehydrogenase (BCKDH) catalyzes a shared step for all 3 branched‐chain alpha‐ketoacids leading to oxidative decarboxylation and CoA thioester formation. (b) BCKDH is composed of E1, E2, and E3 subunits. The E2 transacylase, encoded by *DBT*, forms the core of the complex. Surrounding this core are thiamine pyrophosphate (TPP)‐binding hetero‐tetrameric E1 (E1α/E1β) decarboxylase subunits, encoded by *BCKDHA* and *BCKDHB*. Also surrounding the core are *DLD*‐encoded E3 subunits with lipoamide dehydrogenase function. First, the alpha‐keto acid binds to TPP and is decarboxylated. Second, the lipoyl domain of E2 enters the E1 active site and the branched‐chain is oxidized and transferred to the lipoyl domain as an acyl group. Third, E2 transfers the acyl group to Coenzyme A, leaving behind a reduced lipoyl group. Fourth, E3 oxidizes the lipoyl group to regenerate the cofactor, and FAD is reduced to FADH2. Fifth, FADH2 is oxidized by E3 to FAD+, yielding reduced NADH from NAD+ to complete the cycle

Maple syrup urine disease (MSUD, MIM *248600) is an autosomal recessive inherited metabolic disorder caused by deficiency of the branched‐chain alpha‐ketoacid dehydrogenase enzyme complex (BCKDH). As the second step of the metabolism of the branched‐chain amino acids leucine, isoleucine, and valine, BCKDH catalyzes the oxidative decarboxylation of branched‐chain alpha‐ketoacids and transfer to Coenzyme A. Deficient BCKDH activity leads to a buildup of the branched‐chain amino acids and their respective alpha‐ketoacids, 2‐oxo‐isocaproate (KIC, from leucine), 2‐oxo‐3‐methylvalerate (KMV, from isoleucine), and 2‐oxo‐isovalerate (KIV, from valine) particularly in periods of catabolic stress or high protein load (Figure 1a). Morbidity and mortality in MSUD are principally related to the neurotoxicity of leucine and its alpha‐ketoacid KIC.

BCKDH is a mitochondrial enzyme with three subunits (E1, E2, and E3) that are encoded by 4 genes (Aevarsson et al., 1999). Lack of any of the subunits due to biallelic pathogenic variants in one of the four genes can lead to MSUD. The E1 component is made up of branched‐chain alpha‐ketoacid dehydrogenase E1, alpha (*BCKDHA*, MIM *608348, E.C. 1.2.4.4) and branched‐chain alpha‐ketoacid dehydrogenase E1, beta (*BCKDHB*, MIM *248611). The phosphorylation‐regulated E1 uses a thiamine cofactor to decarboxylate branched‐chain alpha‐ketoacids, releasing carbon dioxide and transferring the resulting branched chain to E2, a lipoamide transacylase. The E2 subunit (*DBT*, MIM *248610, E.C. 2.3.1.168) catalyzes the formation of a branched‐chain Coenzyme A ester. The lipoamide component of E2 is then reset to its oxidized state by dihydrolipoamide dehydrogenase, E3 (*DLD*, MIM *238331, E.C. 1.8.1.4) (Figure 1b). Individuals with disease‐causing variants in both alleles of any of the genes of the E1 or E2 subunits have MSUD referred to as type Ia, type Ib, or type II, depending if the gene involved is *BCKDHA*, *BCKDHB*, or *DBT* respectively. E3 deficiency (aka dihydrolipoamide dehydrogenase deficiency, MIM *264900) has a related but distinct phenotype from MSUD due to the additional use of the E3 subunit in other enzymes. Classic MSUD occurs from the absence or near complete absence of BCKDH activity, while attenuated forms of MSUD are due to the presence of residual enzyme activity (Blackburn et al., 2017).

Classic MSUD presents in the neonatal period with metabolic decompensation manifesting as decreased feeding, vomiting, and lethargy which can progress to coma and death if left untreated and is due to the impact of increased leucine and KIC leading to whole‐brain swelling (Blackburn et al., 2017; Levin et al., 1993; Saudubray et al., 2002; Strauss et al., 2020; Strauss et al., 1993). Even after the neonatal period, individuals remain at risk for additional episodes of decompensation with hyperleucinemia. The imbalances of circulating amino acids may have more subtle impacts on brain structure and function as adolescents and adults with MSUD are at increased risk for ADHD, depression, anxiety, and movement disorders (Carecchio et al., 2011; Muelly et al., 2013).

Hyperleucinemic crisis can be precipitated by large amounts of protein intake or by catabolic states which cause release of endogenous proteins, particularly from muscle, a rich source of leucine. In the acute setting, present therapy focuses on restoring an anabolic state by increasing caloric intake with fat and carbohydrates, by infusion if necessary, and adding insulin if needed. Also essential for therapy are non‐branched‐chain amino acid‐containing metabolic foods (formula), and supplemental isoleucine and valine. In the presence of severe encephalopathy, the addition of hemofiltration has been utilized (Atwal et al., 2015; Aygün et al., 2019; Strauss et al., 1993). Long‐term therapy has been predominately dietary via formula and supplemental isoleucine and valine. It is important to note that this therapy does not prevent metabolic decompensations from occurring, which can be life threatening at any given time point. More recently, liver transplant has been added to the therapeutic options, given the potential to drastically limit if not eliminate the risk of hyperleucinemic crisis, improve chronic biochemical imbalance, and prevent further intellectual impairment (Mazariegos et al., 2011; Strauss et al., 2020; Strauss et al., 1993; Wendel et al., 1999).

**FIGURE 2:**
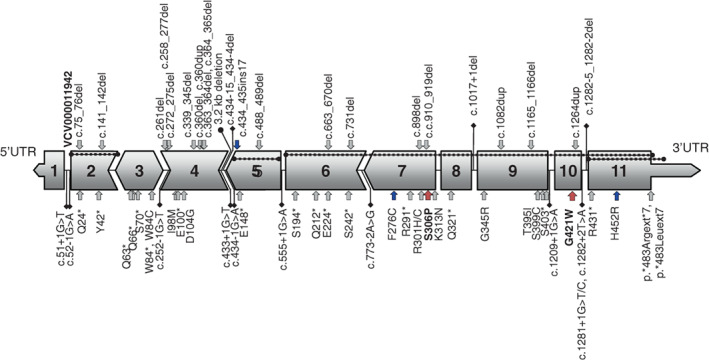
Schematic of *DBT*, annotated with disease‐causing variants: *DBT* has 11 exons with a short 5’ UTR and a long 3’ UTR. Exons 1, 5, 7, 8, 9, and 10 all splice at a codon boundary while introns 2, 3, 4, and 6 splice within a codon with pointed shapes indicating phase. Pathologic variants and likely pathologic variants reported in ClinVar are shown with single nucleotide variants drawn below the gene diagram, and small indels drawn above. Variants reported in ClinVar as leading to thiamine‐responsive MSUD are noted with a dark blue arrow. Pathologic intronic splicing variants are noted with diamond‐headed pins. Large deletions as reported in HGMD are noted with round‐ended beaded lines, except for a large intronic deletion in intron 4 indicated by a round‐headed pin. The missense variants reported in this report are noted in red and label is bolded

MSUD is a rare disorder with an estimated incidence of 1 in 185,000 live births (Nellis et al., 2003, PMID 14567968). The majority of cases in most studies are of type Ia or type Ib MSUD related to pathogenic variants in *BCKDHA* and *BCKDHB*. Within the United States, founder variants in the Mennonite population (NM_000709.4(BCKDHA): c.1312 T > A (p.Tyr438Asn) [VCV000100009.20]) (Fisher et al., 1991a, 1991b) and in persons of Ashkenazi Jewish ancestry (NM_183050.4(BCKDHB):c.548G > C (p.Arg183Pro) [VCV000011937.21]) (Edelmann et al., 2001) make MSUD significantly more common among these populations than in the general population. Worldwide, founder variants have been reported for all three genes *BCKDHA*, *BCKDHB*, and *DBT*. The cases described in this series have type II MSUD caused by two pathogenic variants in *DBT* and suggest the possibility of a founder effect. *DBT* consists of 11 exons (Lau et al., 1991); exons 1,5,7,8,9, and 10 splice at a codon boundary while exons 2, 3, 4, and 6 splice within a codon. The acyltransferase catalytic domain lies within exons 6–11. A variety of disease‐causing variants have been described, including single nucleotide variants, both missense and nonsense, splice‐site variants, deletion‐insertions and deletions, as well as copy number variations (Figure 2).

## 2 MATERIALS AND METHODS

### 2.1 Subjects

Eleven individuals from nine unrelated families with ancestry from El Salvador or Honduras have type II classical MSUD. As a cohort, the average age of presentation was 11 +/− 4 days (Billington et al., 2018). Eight individuals had an abnormal newborn screen (NBS) and were symptomatic upon initial evaluation. One individual had an abnormal NBS but was asymptomatic at the time of confirmatory testing (Case 7). One individual presented with encephalopathy, but the NBS had been improperly collected and therefore was not resulted at time of presentation (Case 10). The final patient had a normal NBS collected at 2 days of life and presented with mild encephalopathy (Case 11). All individuals had a leucine level > 1000 μM (normal <160 μM) at the time of presentation; regardless if presentation was secondary to abnormal NBS or for symptomatic evaluation. See Table 1 for additional information.

**TABLE 1:** Summary of cases of individuals of Central American ancestry with maple syrup urine disease due to variants in *DBT*

Case	Family	Ancestry	DBT Variant 1	DBT Variant 2	NBS +/−	Initial leucine level (μM)	Initial clinical findings (age)	Liver transplant (age)
1	A	El Salvador	DelEx2	DelEx2	+	3028	Encephalopathy and seizure (6d)	Yes (14y)	
2	B	El Salvador	DelEx2	DelEx2	+	2289	Encephalopathy (15d)	No (deceased)	
3					+	1283	Encephalopathy (15d)	Yes (14y)	
4	C	Honduras	DelEx2	DelEx2	+	2016	Encephalopathy (14d)	Yes (4y)	
5	D	Honduras	DelEx2	DelEx2	+	1594	Encephalopathy (5d)	Yes (7 m)	
6	E	El Salvador	DelEx2	DelEx2	+	3200	Encephalopathy (5d)	Yes (4 m)	
7	F	El Salvador	DelEx2	c.916 T > C	+	1016	Asymptomatic (8d)	Yes (15 y)	
8	G	El Salvador	DelEx2	c.916 T > C	+	1336	Encephalopathy (11d)	No	
9	H	Honduras	DelEx2	c.916 T > C	+	1865	Encephalopathy (12d)	Yes (4y)	
10	I	El Salvador	DelEx2	c.916 T > C	(+)	2255	Mild encephalopathy (13d)	No	
11					**−**	2240	Mild encephalopathy (14d)	No	

### 2.2 Methods

#### 2.2.1 Molecular genetic testing

##### Massively parallel sequencing

Genomic DNA was prepared from patient samples using standard clinical lab protocols. Sequencing was performed after library preparation using the TruSight One Exome capture kit (v1.0, Illumina, San Diego, CA) and sequenced using the NextSeq 500 sequencing system with 150 bp paired‐read ends. The DNA sequence was mapped to, and analyzed in comparison with, the published human genome build UCSC hg19 reference sequence in BaseSpace Sequence Hub using BWA Enrichment 2.1.1, GATK v1.6, Enrichment 3.0.0, and Variant Calling Assessment Tool 2.3.0. The targeted coding exons and splice junctions of the known protein‐coding RefSeq genes were assessed for the average depth of coverage of 10X and data quality threshold values of 95%.

Several tools were utilized for variant analysis, including OMIM, HGMD Professional, PubMed, and the data aggregator Alamut Visual v2.11, in addition to numerous databases and algorithms including but not limited to gnomAD v2.0.1, 1000 Genomes Project phase 3, dbSNP, ExAC, ESP, ClinVar, Align GVGD, SIFT, Mutation Taster, PolyPhen, Provean, DANN, FATHMM, and Revel. All sequence variants were classified based on the American College of Medical Genetics and Genomics released standards and guidelines for the interpretation of sequence variants (Richards et al., 2015) and confirmed by independent Sanger sequence analysis. A deletion/duplication in silico algorithm was performed to evaluate for copy number variants (CNV Tool from NextGENe Software v.2.4.1, SOFTGENETICS®, State College, PA) and confirmed by exon‐level array.

##### Sanger sequencing

The relevant portion of the *DBT* gene was PCR‐amplified (AccuPrime™ Taq DNA Polymerase System, ThermoFisher Scientific) from genomic DNA; bidirectional sequence data was obtained and compared to the published reference sequence. For confirmation of delEx2, two primer pairs (PCR1 and PCR2) were designed with amplicons within the deletion and a third pair (PCR3) was designed with M13 tails paired to just outside the deleted region using the NCBI primer designing tool https://www.ncbi.nlm.nih.gov/tools/primer-blast/ and obtained from Integrated DNA Technologies (Figure S1). For c.916 T > C, primers with M13 tails (designed using the ThermoFisher Scientific Primer Designer Tool for PCR & Sanger Sequencing and obtained from Integrated DNA Technologies) were used. Sequencing was performed on an Applied Biosystems™ 3500xL DX CS2 Genetic Analyzer or by GENEWIZ (Azenta Life Sciences) and data was analyzed using Mutation surveyor version 501 (SOFTGENETICS®, State College, PA).

##### Microarray analysis

Total genomic DNA was isolated from peripheral leukocytes and analyzed for copy number changes and areas of homozygosity (AOH) by either the CytoScan™ Dx Assay Kit or the CytoScan™ XON Assay Kit (ThermoFisher Scientific) using the GeneChip® System 3000Dx and analyzed by Chromosome Analysis Suite Dx Software (ChAS Dx Software). The CytoScan™ Dx assay kit was utilized for chromosomal microarray analysis and includes 2.69 million functional markers; 750,000 bi‐allelic single‐nucleotide polymorphism (SNP) probes and 1.9 million non‐polymorphic markers. The XON assay kit was utilized for exon‐level copy number variant analysis and contains 6.55 million copy number probes and 300,000 SNP probes.

#### 2.2.2 Thiamine challenge

A thiamine challenge was performed as part of clinical care for at least two individuals. Although isoleucine is a substrate for the BCKDH enzyme, it is nonetheless relatively well tolerated in individuals with MSUD and indeed is often supplemented as part of their care. A 70 mg/kg isoleucine bolus was given at timepoint 0; blood spots were collected on filter paper at time points 0, 30, 90, 180, and 300 minutes for analysis of branched‐chain amino acids by a liquid chromatography–tandem mass spectrometry method adapted from Tuchman et al. (Tuchman & McCann, 1999). The isoleucine bolus and blood spot analyses were repeated after one week of thiamine supplementation (100 mg).

#### 2.2.3 Liver tissue procurement and isolation of hepatic cells

Hepatocytes were procured from explanted liver through HemoShear Therapeutics and/or Children's National (CNHS Pro0004911) liver procurement program or obtained through QPS Hepatic Biosciences. Hepatocyte isolation from liver tissue obtained from individuals with MSUD was performed at HemoShear Therapeutics, Georgetown University, Children's Hospital of Pittsburgh or QPS Hepatic Biosciences using a protocol previously described by LeCluyse et al (Lecluyse & Alexandre, 2010) and cryopreserved at HemoShear Therapeutics. Cryopreserved control hepatocytes from healthy controls were procured from QPS Hepatic Biosciences.

##### Cell culture and device operating conditions

As described above, primary hepatocytes were obtained and plated in a collagen gel sandwich configuration on the undersurface of the membranes of 75 mm polycarbonate transwells (Corning) using previously described protocols (Dash et al., 2013). The cultures were left overnight in maintenance medium (MM) that consisted of DMEM/F‐12 supplemented with fetal bovine serum (10% at the time of plating). Additionally, the medium contained gentamycin (50 μg/ml), 0.2% ITS (Fisher/MediaTech MT‐25–800CR), and dexamethasone (Cat# D4902, Sigma Aldrich, St. Louis, MO, 1 μM at plating and 250 nM thereafter). On the 2nd day, transwells were set up within HemoShear Therapeutics' hepatocyte bio‐reactor technology in a configuration to allow for control of hemodynamics and transport as previously described (Dash et al., 2013). A proprietary hepatocyte flow medium (HFM), modified from MM but with significantly lower levels of key hormones and growth factors, was continuously perfused on both sides while shear stress was applied on the top surface based on calculations described below. The devices were housed in a controlled environment at 37°C with 5% CO2 mixed with air. Maintenance medium was replaced every 48 hours in non‐flow cultures in a standard CO2 incubator. For all of the flow experiments described in this study, the shear stress of 0.6 dyn/cm2 was used, derived from reference values for the pressure gradient across the sinusoid (ΔP), the radius of sinusoids (r), and the length of the sinusoids (l) obtained from the literature (Dash et al., 2013). The hepatocytes were cultured for 7 days.

For some experiments, primary hepatocytes from individual donors were plated in a collagen gel sandwich configuration on 24‐well or 48‐well plates (Corning) using previously described protocols (Dash et al., 2013). The cultures were incubated in normal or maintenance media that consisted of DMEM/F‐12 supplemented with fetal bovine serum (10% at the time of plating). Additionally, the medium contained gentamycin (50 μg/ml), 0.2% ITS (Fisher/MediaTech MT‐25–800CR), and dexamethasone (Cat# D4902, Sigma Aldrich, St. Louis, MO, 1 μM at plating and 250 nM thereafter). Regular or customized Corning culture media for Hepatocells (Corning, Cat # 354882) was also used for culturing and treatment.

#### 2.2.4 Protein and Western Blot

Samples from one well of a 24‐well plate were harvested using 100 μl of 2X Laemmli sample buffer (BioRad 161–0737) containing beta‐mercaptoethanol (Biorad). Samples were boiled, and subsequently sonicated on ice. Total protein lysates were resolved on a 10% SDS‐PAGE gel and transferred to nitrocellulose. Blots were probed overnight with primary antibody, and at room temperature for 30 minutes with secondary antibody in blocking buffer (LI‐COR 927–40,000). Primary antibodies (1:1000) include: GAPDH (Sigma, G8795), Tubulin (Sigma, T9026), BCKDHA (Thermo Fisher Scientific, PA5‐29300), BCKDHB (Aviva Systems Biology, OAAB00689), DBT (Thermo Fisher Scientific, PA5‐29727), and phospho293 BCKDHA (Bethyl Laboratories, A304‐672A). Secondary antibodies (1:15,000) include: IRDye 800CW Donkey Anti‐Mouse (LI‐COR 926–32,212), IRDye 680LT Donkey Anti‐Rabbit (LI‐COR 926–68,023), and IRDye 800CW Donkey Anti‐Rabbit (LI‐COR 926–32,213). The LI‐COR Odyssey infrared imager was used for image acquisition and the LI‐COR Odyssey Image Studio software was used for densitometry analysis.

#### 2.2.5 Analysis of branched‐chain alpha‐ketoacids

Samples were analyzed by ultrahigh performance liquid chromatography tandem mass spectrometry (UPLC‐MS/MS) using an Agilent 1260 system coupled to a Sciex 6500 triple quadrupole mass spectrometer (Concord, Ontario, L4K 4 V8, Canada). Separation of alpha‐ketoacids was achieved using a flow rate of 0.4 ml × min‐1 using a Luna Omega 1.6um Polar C18 50x2.1 00B‐4748‐AN column temperature controlled at 40°C. A linear gradient of mobile phase A (0.05% formic acid [v/v]) and mobile phase B (methanol) were used. Briefly, 5% mobile phase B for 0.5 min followed by a linear gradient to 10% B for 4 min. The column was washed with 95% mobile phase B for 1.2 min and then re‐equilibrated for 3 min. Electrospray ionization parameters were optimized using 20% methanol at a flow rate of 0.4 ml per minute in negative ion mode. Ion spray −4500 V, GS1 and GS2 30 L per minute, curtain gas 30, source and gas temperature 550°C, CAD gas −2, and EP ‐10. Collison energy was individually optimized for each analyte. Ion monitoring 0.5 amu for Q1 resolution and 0.5 amu for Q3 resolution with the following selected reaction monitoring pairs (m/z) for each alpha‐ketoacid and its internal standard (IS): KIC 129 > 85, KIC‐IS 131.9 > 87.9, KMV 129 > 85, KMV‐IS 137 > 92.9, KIV 115 > 71, KVI‐IS 120 > 74.9. The method allows for separation and detection with the upper limit of detection in the 10–6 mM range.

## 3 RESULTS

### 3.1 Clinical case summaries

Table 1 summarizes the 11 cases with type II classical MSUD due to either homozygous delEx2 or compound heterozygous delEx2;c.916 T > C variants in *DBT* presented in this series. At the time of this publication, seven individuals have undergone a liver transplant due to concern for recurrent hyperleucinemic crises; five were transplanted in childhood or adolescence. Two cases were incredibly difficult to manage by diet alone and as a result were transplanted in infancy. One individual died during a metabolic decompensation in adolescence.

Case 1 is a male of Salvadoran ancestry who presented due to an abnormal NBS and was found to have an elevated leucine level of 3028 μM on day of life (DOL) 6. He had a seizure immediately following presentation and was placed on hemodialysis. Liver transplant was performed at age 14 years for improved metabolic stability.

Case 2 is a male of Salvadoran descent, who is also a twin to case 3. He presented with an encephalopathic crisis at DOL 15; his NBS was positive and initial leucine level was 2289 μM. A trial of thiamine supplementation (50 mg/kg) was done in infancy but was discontinued after no apparent response. He is now status post liver transplant, done at 14 years for metabolic stability.

Case 3 is a deceased male of Salvadoran descent, and twin to case 2. His initial leucine level was 1283 μM and he also underwent a trial of thiamine supplementation (50 mg/kg) in infancy. He had several decompensation events including a fatal cerebral herniation at the age of 14 years.

Of note, an older sibling to cases 2 and 3 passed away in the neonatal period. Although no diagnosis was made, the circumstances of her demise are compatible with MSUD.

Case 4 is a male of Honduran ancestry who presented with encephalopathy on DOL 14 with an elevated leucine level of 2016 μM and was placed on dialysis. In hindsight, his NBS had been abnormal. He is now status post liver transplant.

Case 5 is a male of Honduran ancestry who presented with encephalopathy and hyperleucinemia on DOL 5; simultaneously, his NBS returned as abnormal. His initial leucine level was 1594 μM and he was noted to have a full fontanelle and tachypnea on physical exam prompting initiation of hemodialysis. A chromosomal microarray was obtained due to his rapid deterioration. He underwent a liver transplant at 7 months for stability reasons and resultant hepatocytes were available for analysis.

Case 6 is a female of Honduran descent who presented on DOL 5 because of an abnormal NBS. On evaluation, she was noted to be encephalopathic with a leucine level of 3200 μM. During infancy, her leucine levels were difficult to manage with dietary treatment alone and she underwent a liver transplant at 4 months of age.

Case 7 is a female of Salvadoran ancestry who presented due to an abnormal NBS on DOL 8 with an elevated leucine level of 1016 μM. Fibroblast BCKDH activity was noted to be 0.6% of normal and consistent with classical MSUD (proband activity 52 +/− 30 nmol/min/mg protein, unaffected control activity 9225 +/− 378, MSUD control activity 0 +/− 0). A thiamine challenge was performed as described in this report and was not beneficial. She is status post liver transplantation at age 15 years for increased stability and improved leucine control while allowing liberalization of diet.

Case 8 is a female of Salvadoran ancestry who presented on DOL 11 for an abnormal NBS. She was encephalopathic at presentation with a leucine of 1336 μM and required hemodialysis. She has had several admissions for metabolic decompensation and has declined liver transplantation.

Case 9 is a male of Honduran ancestry who presented on DOL 12 in hyperleucinemic crisis with a leucine level 1865 μM. His NBS was abnormal, and he underwent hemodialysis as a neonate. A thiamine challenge was performed as described in this report and was not favorable. He underwent an orthotopic liver transplant at age 4 years for improved stability. Resultant hepatocytes were available for analysis.

Case 10 is a male of Salvadoran descent who presented on DOL 12 with poor feeding and irritability that progressed to encephalopathy and bicycling on DOL 13. His initial leucine level was 2255 μM. The NBS had been improperly collected and therefore did not result; however, a second NBS collected on DOL 13 was abnormal. Liver transplantation was declined, and he continues on dietary therapy. His parents were available for genetic testing to facilitate phasing of the *DBT* variants.

Case 11 is a female of Salvadoran descent and sibling to Case 10. She was born following pre‐conceptional genetic counseling where prenatal screening and IVF with preimplantation genetic screening were declined. Plasma amino acids collected at 23 hours of life revealed a leucine level of 190 μM and NBS collected at 32 hours of life was negative. She presented on DOL 14 with characteristic odor of maple syrup in cerumen and urine, and mild encephalopathy; her leucine level was 2240 μM. Liver transplantation was declined, and she continues on dietary therapy.

### 3.2 Molecular findings

#### 3.2.1 NM_001918.3(DBT):C.48_171del [VCV000011942.1]

Cases 1–6 are homozygous for a deletion of Exon 2 (delEx2). This variant was detected by in silico structural variant prediction algorithms using next‐generation sequencing data, by exon‐level array (case 1, [GRCh38] NC_000001.11:g.100239987_100241333del, Figure S1), and by Sanger sequencing (case 7, NC_000001.11:g.100239099_100246416del, Figure S2). A deletion of exon 2, both in the homozygous and compound heterozygous state with another variant, has been previously reported in the literature; homozygous delEx2 was detected in an individual with MSUD by targeted comparative genomic hybridization array (NC_000001.11:g.100238906_100246406del) (Herring et al., 1991; Tayeh et al., 2009). It is interesting to note that there is a 7 base pair (TGTACTG) microhomologous region in intron 1 and intron 2 identified on either end of the deletion suggesting a possible mechanism (Ankala et al., 2012). The deletion of exon 2 is classified as pathogenic in ClinVar (Accession # VCV000011942.1), although functional evidence has not been ascertained. Loss of exon 2 introduces a frameshift for the remainder of the transcript leading to early termination since the first and second splice junctions are out of phase (Figure 2); hence, this change would also be predicted to lead to nonsense‐mediated decay. Furthermore, the deletion is not found in 141,456 individuals from the Genome Aggregation Database (gnomAD) indicating that it is rare in the sampled general populations. Given all of this information, the delEx2 variant was classified as pathogenic.

#### 3.2.2 NM_001918.5(DBT):C.916 T > C (p.Ser306Pro) [VCV001342860.1]

Cases 7–11 are compound heterozygous for delEx2 and c.916 T > C. The c.916 T > C variant was detected by massively parallel sequencing and confirmed by Sanger sequencing (Figure S3). Serine at position 306 is a highly conserved amino acid (considering 12 species, including vertebrates as well as *C. elegans*) and there is a moderate physiochemical difference between serine and proline resulting from the change of a highly conserved polar uncharged amino acid to a hydrophobic amino acid. Computational predictors varied in their assessment of the c.916 T > C variant. For example, MutationTaster, SIFT, Provean, and DANN all suggest the variant is disease‐causing, while FATHMM and Revel predict the change to be benign. The CADD (hg38 v1.6) score for this variant is 23.6 (Rentzsch et al., 2021). The c.916 T > C variant has been reported only once before in the literature (Park et al., 2016), as a variant of unknown significance, in a heterozygous state, and solely as an incidental finding in an individual from Korea who does not have MSUD and instead has phenylketonuria due to known pathogenic *PAH* variants. Finally, the variant is absent from gnomAD and therefore is rare in the general population. Given all of this information, the c.916 T > C variant was initially classified as a variant of uncertain significance.

#### 3.2.3 Phasing of the delEx2 and c.916 T > C variants

In order to assess phasing of the delEx2 and c.916 T > C variants, parental samples were collected from the mother and father of cases 10 and 11. In this family, delEx2 was paternally inherited and c.916 T > C was maternally inherited (Figure S3), confirming these variants are in *trans* and that compound heterozygosity for the two variants co‐segregates within the family with affected status.

#### 3.2.4 Investigation for the possibility of a founder effect

A founder effect variant would be characterized by identity by descent in the chromosomal region surrounding the variant of interest, as part of a haplotype block.

For Case 5, chromosomal microarray identified a 16.7 Mb AOH surrounding the *DBT* locus on chromosome 1 (arr[hg19] 1p22.1p13.3(93,953,486‐110,677,023)x2 hmz) (Figure S4a); the lack of identification of additional AOH regions in this case is consistent with non‐consanguinity of the parents.

To further assess a founder effect hypothesis, chromosome 1 variant call files for two unrelated individuals with MSUD who are compound heterozygous (delEx2;c.916 T > C) were compared to other cases in the laboratory database. The calls were assessed at each point for homozygous reference allele, heterozygous, or homozygous alternate allele to determine if they were the same or discrepant. For the ~15 Mb area surrounding the *DBT* locus, the two unrelated individuals with MSUD had similar variant calls suggesting identity by descent for both *DBT* alleles inherited by these two individuals (Figure S4b).

### 3.3 Thiamine supplementation with isoleucine challenge

Previous in vitro studies by others suggest that missense variants in *DBT* may be responsive to thiamine, showing enhanced enzyme activity and ameliorated phenotype with thiamine supplementation (Fernhoff et al., 1985). At least two missense variants in *DBT* are thiamine responsive (NM_001918.5(*DBT*)):c.827 T > G (p.Phe276Cys) [VCV000011943.13] (C. W. Fisher et al., 1991a; 1991b) and NM_001918.5(*DBT*):c.1355A > G (p.His452Arg) [VCV000011952.1] (Chuang et al., 2004) (Figure 2). Given the reports of thiamine responsiveness in potentially similar situations to this case series, a thiamine challenge was undertaken for cases 7 and 9 to determine if those with the c.916 T > C variant might benefit from thiamine supplementation. Given the similar isoleucine levels with and without thiamine supplementation, it was concluded that thiamine supplementation did not affect in vivo BCKDH activity for these cases (Figure S5).

### 3.4 Functional analysis of *DBT* variants

**FIGURE 3:**
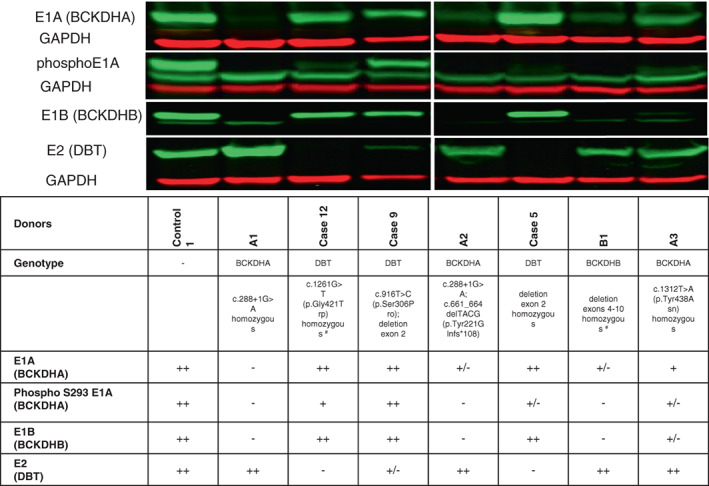
Western Blot of seven individuals with MSUD and one control case demonstrating expression of the subunits of BCKDH including E1A, phosphorylated E1A, E1B, E2, and the loading control GAPDH. Results are summarized in the table below the image and include genotype of donor for each hepatocyte cell line

Explanted liver was obtained from Cases 5 (homozygous delEx2) and 9 (compound heterozygous delEx2;c.916 T > C). HemoShear Therapeutics' hepatocyte bioreactor system was utilized to maintain normal hepatocyte structure and function (Figure S6) (Chapman et al., 2016; Collado et al., 2020). The protein expression of each of the 3 subunits (E1 alpha, E1 beta, E2 (DBT)) and phosphorylation state of E1A was analyzed by Western Blot (Figure 3). For controls, livers from individuals with disease‐causing variants in *BCKDHA* (A1, A2, A3) or *BCKDHB* (B1) in addition to an individual homozygous for another missense variant in *DBT* (Case 12, NM_001918.5:c.1261G > T[p.Gly421Trp] [VCV000203669.2]) as well as an unaffected individual were used; see supplemental material for clinical histories, variant details, and discussion of pathogenicity. Results of the homozygous delEx2 hepatocytes demonstrated neither DBT expression nor activity; whereas, hepatocytes compound heterozygous for delEx2;c.916 T > C have a small amount of DBT detected.

### 3.5 In vitro branched‐chain alpha‐ketoacid analysis

**FIGURE 4:**
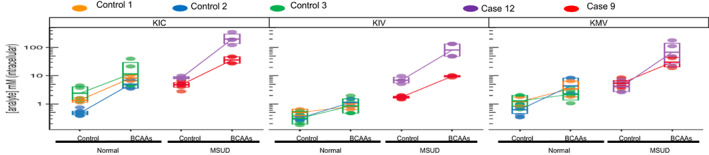
Intracellular levels (mM) of 2‐oxocaproate (KIC from leucine), 2‐oxo‐isovalerate (KIV from valine) and 2‐oxo‐3‐methylvalerate (KMV from isoleucine) as measured by UPLC‐MS/MS following growth in control media (Hepatocell) and Hepatocell supplemented with 5 mM leucine, 5 mM valine, and 5 mM isoleucine in three control hepatocyte lines with presumable normal biochemistry and 2 hepatocyte lines from individuals with MSUD due to variants in *DBT* (cases 9 and 12)

To evaluate the biochemical function of *DBT* variants under controlled conditions, branched‐chain alpha‐ketoacids were measured by UPLC‐MS/MS as described above. KIC, KMV, and KIV levels were determined for unaffected liver cells and hepatocytes from those with MSUD (i.e., delEx2 homozygote) under standard culture conditions (control) in addition to a branched‐chain amino acid (BCAA) challenge by adding 5 mM of leucine, isoleucine, and valine to the culture medium (Figure 4). Increased levels of KIC, KIV, and KMV were noted in the hepatocytes of individuals with *DBT* variants. In general, levels of KIV were slightly less than KIC or KMV in most conditions; KIC appears to accumulate more intracellularly than KIV and KMV, especially after the BCAA challenge. Moreover, the BCAA challenge increased branched‐chain alpha‐ketoacid levels 15‐22‐fold for hepatocytes that had no enzyme detected by Western Blot, compared to unaffected cells with a 1‐fold increase in branched‐chain alpha‐ketoacid levels following the BCAA challenge.

## 4 DISCUSSION

Prior to this study, the delEx2 variant had been reported in only a handful of case reports. With this case series, delEx2 is now described in multiple unrelated individuals, both in a homozygous and a compound heterozygous state. Of the six cases from five unrelated families with homozygous delEx2, all but one individual has undergone liver transplant due to the severity of their disease, and that one individual succumbed to a fatal cerebral herniation in adolescence. With regard to its biochemical characterization, there does not appear to be any protein expression in hepatocytes homozygous for delEx2. Furthermore, this variant is absent from population databases. In summary, the data described in this study affirms and contributes to the classification of this variant as pathogenic by applying the 2015 ACMG/AMP criteria PVS1 + PS3 + PM2 + PP5.

There is little in the medical literature regarding the c.916 T > C variant with the exception of an incidental finding in the heterozygous state in an unaffected individual. In this case series, the c.916 T > C variant was observed with delEx2 in five cases from four unrelated families and confirmed to be in *trans* with biparental inheritance in one family. Additionally, fibroblast BCKDH activity was noted to be 0.6% of normal in one individual and consistent with classical MSUD. Individuals who are compound heterozygous for delEx2;c.916 T > C appear to present slightly later (DOL 8–14) than expected for classic MSUD (typically DOL 5), albeit they still present in the neonatal period with markedly elevated leucine levels >1000 μM and encephalopathy. The clinical course of these individuals is also consistent with classic MSUD with multiple episodes of metabolic decompensation and unresponsiveness to thiamine. Four individuals have required hemodialysis for an encephalopathic crisis at some point and two individuals have undergone liver transplantation for metabolic stability. Biochemically, protein expression was profoundly reduced in hepatocytes from one of these cases, which also showed increased accumulation of intracellular branched‐chain alpha‐ketoacids with a BCAA challenge. In general, missense variants in *DBT* are a known cause of disease. Moreover, several other missense variants in close proximity to c.916 T > C that also occur in exon 7 and within the acyltransferase catalytic region are known to be disease‐causing (Figure 2). Lastly, and similarly to the delEx2 variant, the c.916 T > C variant is absent from population databases. Employing the ACMG/AMP criteria PS3 + PM2 + PM3 + PP1 + PP2 + PP3, the c.916 T > C variant is pathogenic.

There are some caveats to consider when applying the ACMG/AMP scoring criteria to the delEx2 and c.916 T > C variants. For example, it is important to note that individuals of Central American ancestry may not have been well sampled in the large population databases and, as a result, the absence of these variants may be of less significance for this population. A disadvantage of using population databases is that not all genotypes are annotated, in part due to the lack of diversity within the databases. For this reason, it is possible that other potentially pathogenic variants that occur with a high frequency in a population (e.g., a founder effect variant impacting risk for carrier status) may not be identified. It is no small task to expand access to testing to populations that are under‐served, under‐represented, and/or impoverished, but as a field this must be done.

Another drawback for this study was the limitation (in terms of material) in which to assay the MSUD pathophysiology. Hepatocytes, when isolated, have differing abilities to grow following a long‐term freeze which impacts amounts available for various assays; furthermore, hepatocytes may not be the best cell type for studying MSUD due to organ‐to‐organ differences in enzyme function (Kun et al., 1966; Kun & Volfin, 1966). Finally, although the biochemical data presented here indicates reduced or absent protein levels in liver cells derived from individuals with MSUD as well as reduced BCKDH activity (represented by elevated branched‐chain alpha‐ketoacids), it cannot be completely ruled out that the variants under investigation are the cause of these biochemical features without a transgenic experiment attempting to rescue a *DBT* null cell type with a genetic construct that includes these variants. This experiment would eliminate the possibility of an as‐yet cryptic variant in linkage with the reported variants being responsible for the observed biochemistry. Not only is this type of experiment beyond the scope of investigation for this report, it would be extremely unlikely that five subjects from four unrelated families would have an unidentified, non‐coding variant in linkage with c.916 T > C. Despite these limitations, the evidence of pathogenicity is still quite strong for delEx2 and c.916 T > C.

It is interesting to note that this cohort does not include an individual homozygous for the c.916 T > C variant. This may be due to either a less severe enzyme deficiency preventing ascertainment (there appears to be some detectable protein and some residual function even with the delEx2 variant in the compound heterozygous state) or less likely, homozygosity for the c.916 T > C variant produces a severe enzymatic defect that is lethal. Perhaps, and most likely, the c.916 T > C variant is significantly less common in the population and thus, less likely to be seen in a homozygous state. Until an individual is identified to be homozygous for the c.916 T > C variant, the phenotype can only be speculated.

The observation that all of the individuals in this case series have ancestors from El Salvador or adjacent Honduras is interesting. It is also worth noting that within the United States, the Washington, DC, metro area has one of the largest percentages of immigrants from El Salvador. The estimated population of persons of Central American ancestry in the Washington, DC, metro area is 387,000 out of 6.3 million people (US Census American Communities Survey 2019 data). With the eleven cases presented here, the prevalence rate appears to be around 1 in 35,000 for this population. For the delEx2 and c.916 T > C variants, the carrier rate can also be estimated at 1 in 94 persons of Salvadoran or Honduran ancestry. It is possible that other US cities with large Central American populations (e.g., Houston, Los Angeles, Miami, New Orleans, New York) as well as the nations of El Salvador and Honduras may also have individuals with MSUD due to these variants in *DBT*. Furthermore, they may also have an increased number of cases, if these variants represent a founder effect. In fact, some initial data from individuals in this case series suggest this possibility as haplotype blocks of identity by descent were noted surrounding the *DBT* locus. Additional data are needed to better assess this hypothesis and could allow for estimation of the geographic and historical origins of this variant. Analysis and comparison of additional genomic data within the cohort and collection of family history including more precise geographic origins within countries of ancestry would be interesting.

A major challenge in studying pathogenicity is identifying models that best recapitulate the disease state. There are few reliable methods to study MSUD biology in the laboratory because of organ‐to‐organ differences in enzyme function (Al Dhahouri et al., 2018; Kun et al., 1966; Kun & Volfin, 1966) as well as differences in animal models compared to human. In this study, models were developed using hepatocytes obtained from explanted livers of individuals with MSUD, where the relevant genetic information remains intact. Although primary hepatocytes are challenging to procure and maintain in culture, they afford the most faithful representation of liver metabolism and the individual's original genotype. Success in this approach is dependent on three crucial factors: (1) access to explanted tissue; (2) timely isolation of hepatocytes; and (3) ability to maintain hepatocytes in a functionally differentiated state in culture. In individuals with MSUD, liver transplantation is performed for those with frequent metabolic decompensations. In collaboration with other pediatric liver transplantation centers in the US, a protocol has been established in order to gain IRB‐approved access to explanted livers from individuals with MSUD. Livers identified for isolation are procured from the surgical team and perfused within 30 minutes of organ removal, with concomitant timely isolation and cryopreservation of primary hepatocytes. Using the HemoShear Therapeutics' hepatocyte bioreactor, in vivo‐like morphology and functionality is accurately reproduced, and the metabolic disorder is restored, permitting further experimentation. Most importantly, levels of the branched‐chain alpha‐ketoacids KIC, KIV, and KMV can be correlated with BCKDH expression and activity using this hepatocyte system. Patient‐derived MSUD organotypic models may offer utility and relevance to understand MSUD liver biology as well as to use for discovery and development of novel therapeutics.

## 5 CONCLUSION

In summary, this case series describes eleven individuals from nine unrelated families with MSUD caused by pathogenic variants in *DBT*; six homozygotes for delEx2 and five compound heterozygotes for delEx2;c.916 T > C. All individuals presented with marked elevation of leucine >1000 μM in the neonatal period, consistent with a type II classical MSUD phenotype. Neither those homozygous for delEx2 nor compound heterozygous for delEx2;c.916 T > C are responsive to thiamine. Seven individuals are status post liver transplant, with two individuals requiring transplantation in infancy secondary to volatile leucine levels refractory to dietary management. Western Blot and measurement of branched‐chain alpha‐ketoacids in explanted liver cells demonstrated little to no protein product in addition to accumulation of KIC, KMV, and KIV under both standard culture conditions and with a BCAA challenge. Although delEx2 has been previously reported, this study further supports the pathogenicity of this variant by providing functional data. The c.916 T > C variant has not been well‐described prior to this study and the interrogation of this variant by this study substantiates that it is a disease‐causing variant. Finally, these two variants have been identified in a number of unrelated individuals originating from Honduras or El Salvador and additional studies are warranted to evaluate for the possibility of a founder effect.

## AUTHOR CONTRIBUTIONS

CJB, KAC, MSC, and KCO designed the study, reviewed the data, and participated in the writing and revision of the manuscript. EL participated in data collection. BWM and SB participated in the molecular analysis portions of the study and generation of Figures. CW participated in curation of explanted livers. MO, RAF, SAH, BRW, and MSC participated in the design and analysis of studies using the HemoShear Therapeutics' hepatocyte bio‐reactor.

## CONFLICT OF INTEREST

Employees of HemoShear Therapeutics are designated in the author list. Otherwise, no other authors have conflict of interest.

## Supporting information


**Figure S1** Exon targeted array data indicating reduced hybridization for exon 2 probes. Control sample shows Exon 2 probes clustering around Log2 ratio of 0 indicating normal copy number. Case 10 and case 1 show progressively less exon 2 probe binding indicative of heterozygous and homozygous deletion of exon 2, respectively.
**Figure S2**: Sanger sequencing confirmation of *DBT* delEx2 variant detected by next‐generation sequencing
**Figure S3**: Parental testing to determine phase indicates c.916 T > C in trans with deletion of exon 2 in Case 10. Sanger sequencing of Case 10 and his mother and exon targeted array for Case 10 and his father are shown indicating inheritance of each variant from a separate parent. For Sanger sequencing, the following primers were used
**Figure S4**: Possible area of identity by descent
**Figure S5**: Branched‐chain amino acid analysis via dried blood spot at time points 0, 30, 90, 180, and 300 minutes following a 70 mg/kg isoleucine bolus at baseline and repeated after >1 week of thiamine supplementation (B1, 100 mg).
**Figure S6:** (a) Sketch of HemoShear Therapeutics' hepatocyte bio‐reactor. Medium is infused from the infusion port and goes through the upper chamber with the cone (orange triangle which spins) providing for down‐ward force on the apical side of the hepatocytes in addition to perfusion through the lower chamber providing horizontal force along the basal side of the hepatocytes. Out‐flow is collected by the transport outflow. In these studies, cells are exposed to normal Hepatocell medium as well as Hepatocell medium enhanced with additional 5 mM leucine, 5 mM isoleucine, and 5 mM valine to recapitulate a decompensation event. (b) Images of hepatocytes in the HemoShear Therapeutics' hepatocyte bio‐reactor. Slides demonstrate normal structure by immunostaining.

**Appendix S1** Supporting Information

## Data Availability

The data that support the findings of this study are available from the corresponding author upon reasonable request.
