# Supplementary material for: Genomic and biochemical analysis of repeatedly observed variants in DBT in individuals with maple syrup urine disease of Central American ancestry
Source: Am J Med Genet A. 2022 Jul 7;188(9):2738–49. doi: 10.1002/ajmg.a.62893 (PMC9542135; doi:10.1002/ajmg.a.62893)
Supplement: Supplementary file 1 — Figure S1 Exon targeted array data indicating reduced hybridization for exon 2 probes. Control sample shows Exon 2 probes clustering around Log2 ratio of 0 indicating normal copy number. Case 10 and case 1 show progressively less exon 2 probe binding indicative of heterozygous and homozygous deletion of exon 2, respectively. Figure S2: Sanger sequencing confirmation of DBT delEx2 variant detected by next‐generation sequencing Figure S3: Parental testing to determine phase indicates c.916 T > C in trans with deletion of exon 2 in Case 10. Sanger sequencing of Case 10 and his mother and exon targeted array for Case 10 and his father are shown indicating inheritance of each variant from a separate parent. For Sanger sequencing, the following primers were used Figure S4: Possible area of identity by descent Figure S5: Branched‐chain amino acid analysis via dried blood spot at time points 0, 30, 90, 180, and 300 minutes following a 70 mg/kg isoleucine bolus at baseline and repeated after >1 week of thiamine supplementation (B1, 100 mg). Figure S6: (a) Sketch of HemoShear Therapeutics' hepatocyte bio‐reactor. Medium is infused from the infusion port and goes through the upper chamber with the cone (orange triangle which spins) providing for down‐ward force on the apical side of the hepatocytes in addition to perfusion through the lower chamber providing horizontal force along the basal side of the hepatocytes. Out‐flow is collected by the transport outflow. In these studies, cells are exposed to normal Hepatocell medium as well as Hepatocell medium enhanced with additional 5 mM leucine, 5 mM isoleucine, and 5 mM valine to recapitulate a decompensation event. (b) Images of hepatocytes in the HemoShear Therapeutics' hepatocyte bio‐reactor. Slides demonstrate normal structure by immunostaining. [file AJMG-188-2738-s002.pptx]

## Slide 1
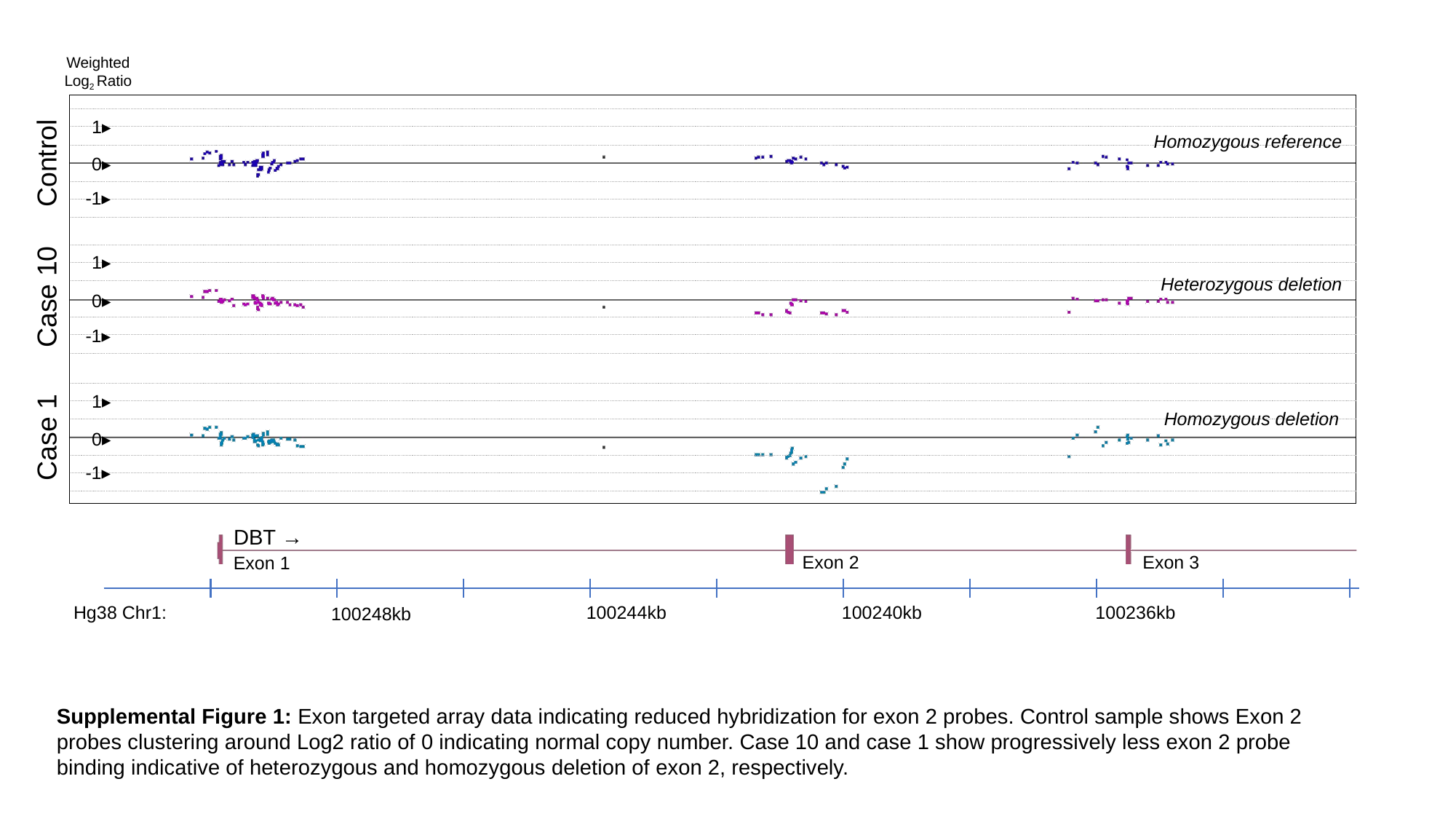

Weighted Log2 Ratio
1▸
Homozygous reference
Control
0▸
-1▸
1▸
Heterozygous deletion
Case 10
0▸
-1▸
1▸
Homozygous deletion
Case 1
0▸
-1▸
DBT →
Exon 3
Exon 2
Exon 1
Hg38 Chr1:
100244kb
100240kb
100236kb
100248kb
Supplemental Figure 1: Exon targeted array data indicating reduced hybridization for exon 2 probes. Control sample shows Exon 2 probes clustering around Log2 ratio of 0 indicating normal copy number. Case 10 and case 1 show progressively less exon 2 probe binding indicative of heterozygous and homozygous deletion of exon 2, respectively.

## Slide 2
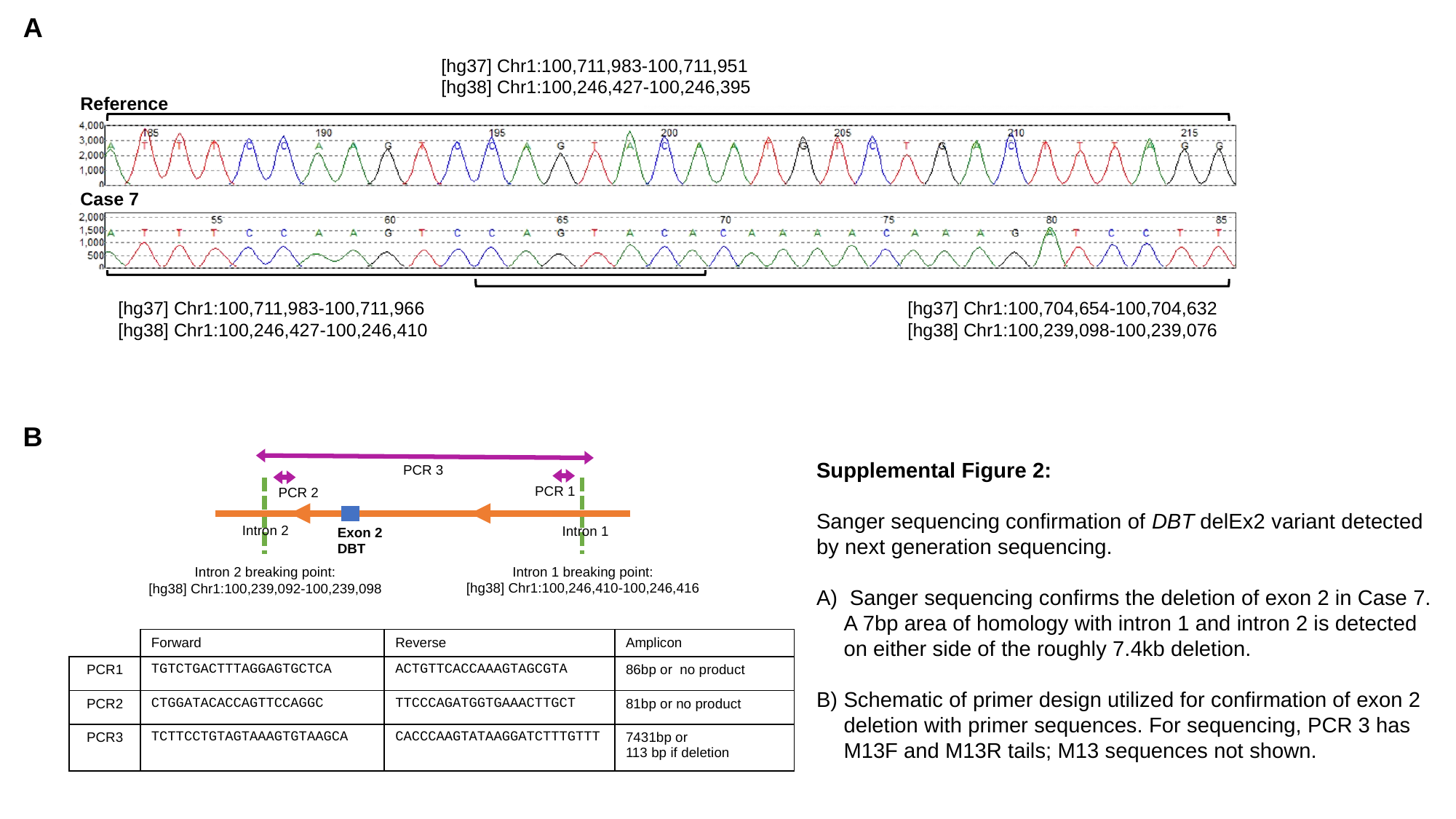

A
[hg37] Chr1:100,711,983-100,711,951
[hg38] Chr1:100,246,427-100,246,395
Reference
Case 7
[hg37] Chr1:100,711,983-100,711,966
[hg38] Chr1:100,246,427-100,246,410
[hg37] Chr1:100,704,654-100,704,632
[hg38] Chr1:100,239,098-100,239,076
B
Supplemental Figure 2:
Sanger sequencing confirmation of DBT delEx2 variant detected by next generation sequencing.
 Sanger sequencing confirms the deletion of exon 2 in Case 7. A 7bp area of homology with intron 1 and intron 2 is detected on either side of the roughly 7.4kb deletion.
Schematic of primer design utilized for confirmation of exon 2 deletion with primer sequences. For sequencing, PCR 3 has M13F and M13R tails; M13 sequences not shown.
PCR 3
PCR 1
PCR 2
Intron 2
Intron 1
Exon 2
DBT
Intron 1 breaking point:
[hg38] Chr1:100,246,410-100,246,416
Intron 2 breaking point:
[hg38] Chr1:100,239,092-100,239,098
| | Forward | Reverse | Amplicon |
| --- | --- | --- | --- |
| PCR1 | TGTCTGACTTTAGGAGTGCTCA | ACTGTTCACCAAAGTAGCGTA | 86bp or no product |
| PCR2 | CTGGATACACCAGTTCCAGGC | TTCCCAGATGGTGAAACTTGCT | 81bp or no product |
| PCR3 | TCTTCCTGTAGTAAAGTGTAAGCA | CACCCAAGTATAAGGATCTTTGTTT | 7431bp or 113 bp if deletion |

## Slide 3
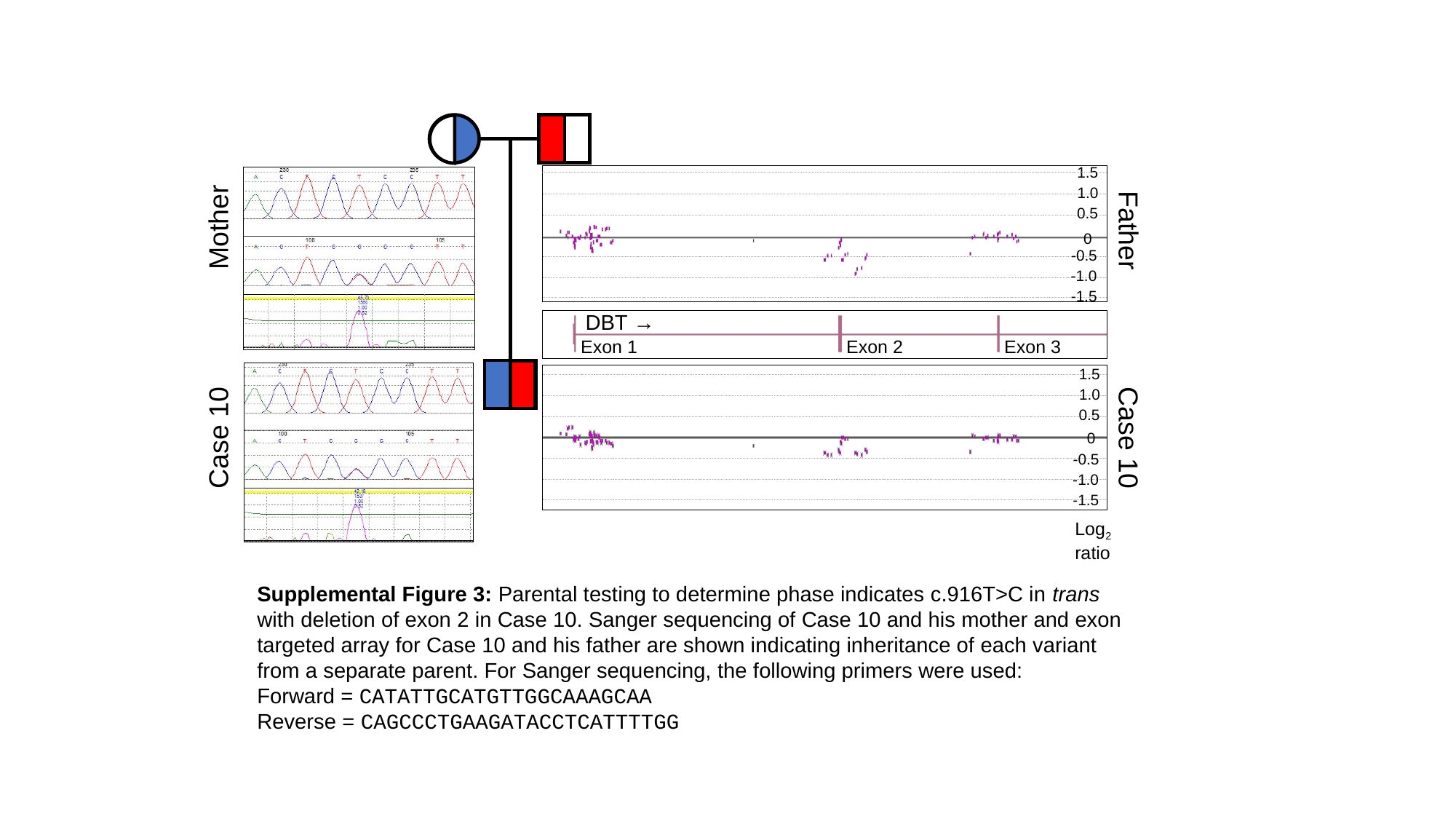

1.5
1.0
0.5
Mother
Father
0
-0.5
-1.0
-1.5
DBT →
Exon 1
Exon 2
Exon 3
1.5
1.0
0.5
Case 10
Case 10
0
-0.5
-1.0
-1.5
Log2
ratio
Supplemental Figure 3: Parental testing to determine phase indicates c.916T>C in trans with deletion of exon 2 in Case 10. Sanger sequencing of Case 10 and his mother and exon targeted array for Case 10 and his father are shown indicating inheritance of each variant from a separate parent. For Sanger sequencing, the following primers were used:
Forward = CATATTGCATGTTGGCAAAGCAA
Reverse = CAGCCCTGAAGATACCTCATTTTGG

## Slide 4
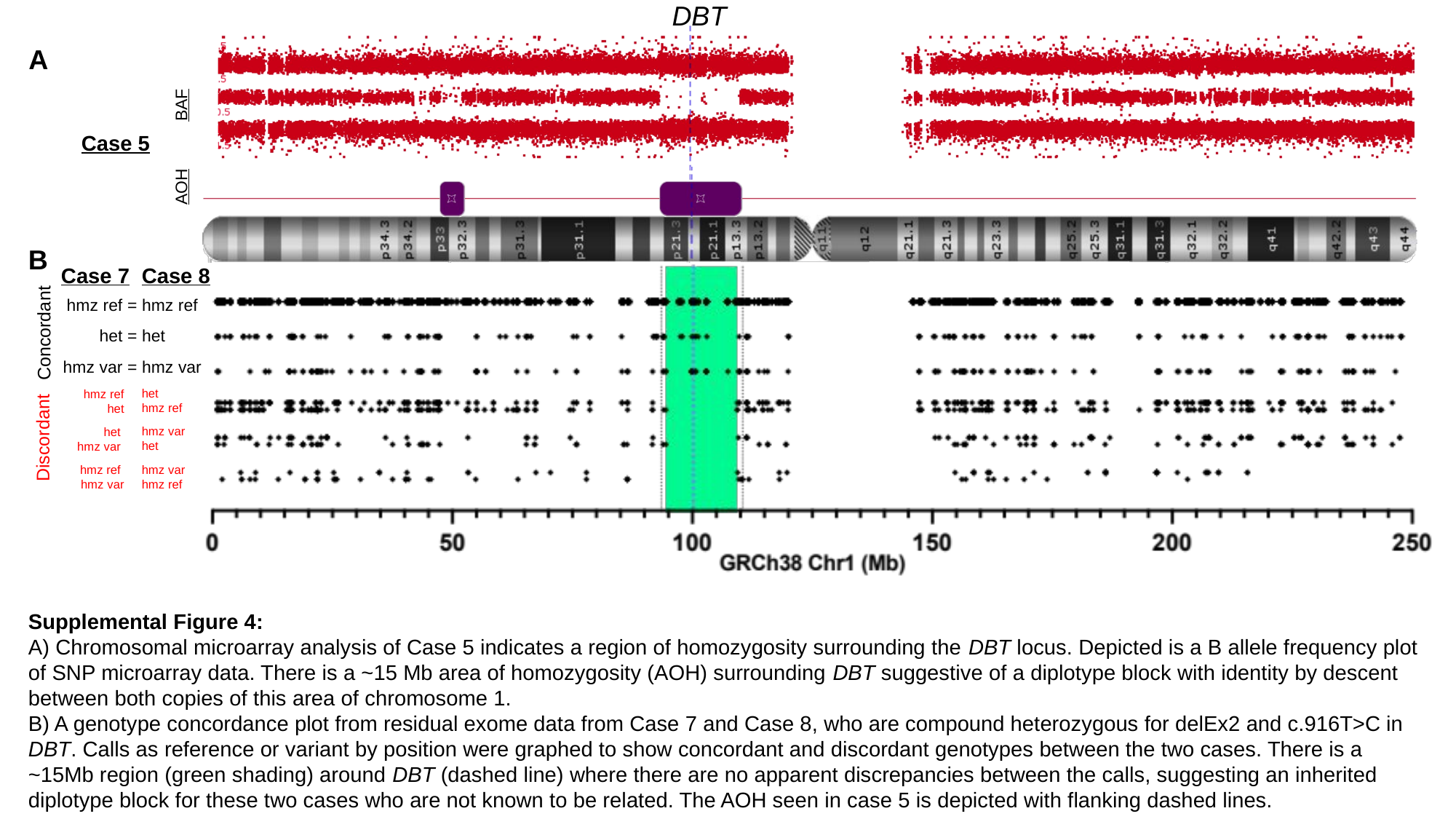

DBT
A
BAF
Case 5
AOH
B
Case 7
Case 8
hmz ref = hmz ref
het = het
hmz var = hmz var
Concordant
het
hmz ref
hmz var
het
hmz var
hmz ref
hmz ref
het
het
hmz var
hmz ref
hmz var
Discordant
Supplemental Figure 4:
A) Chromosomal microarray analysis of Case 5 indicates a region of homozygosity surrounding the DBT locus. Depicted is a B allele frequency plot of SNP microarray data. There is a ~15 Mb area of homozygosity (AOH) surrounding DBT suggestive of a diplotype block with identity by descent between both copies of this area of chromosome 1.
B) A genotype concordance plot from residual exome data from Case 7 and Case 8, who are compound heterozygous for delEx2 and c.916T>C in DBT. Calls as reference or variant by position were graphed to show concordant and discordant genotypes between the two cases. There is a ~15Mb region (green shading) around DBT (dashed line) where there are no apparent discrepancies between the calls, suggesting an inherited diplotype block for these two cases who are not known to be related. The AOH seen in case 5 is depicted with flanking dashed lines.

## Slide 5
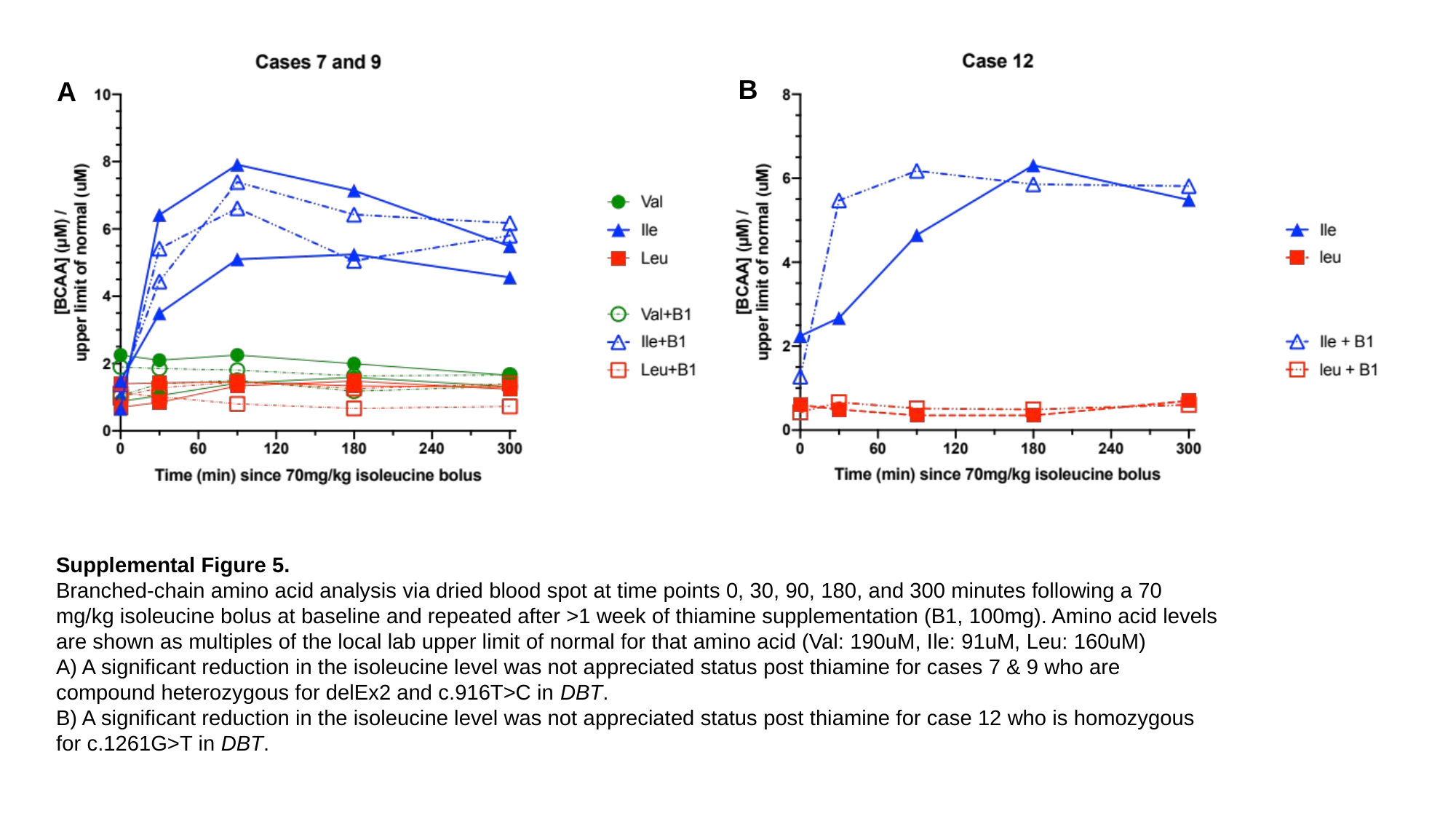

B
A
Supplemental Figure 5.
Branched-chain amino acid analysis via dried blood spot at time points 0, 30, 90, 180, and 300 minutes following a 70 mg/kg isoleucine bolus at baseline and repeated after >1 week of thiamine supplementation (B1, 100mg). Amino acid levels are shown as multiples of the local lab upper limit of normal for that amino acid (Val: 190uM, Ile: 91uM, Leu: 160uM)
A) A significant reduction in the isoleucine level was not appreciated status post thiamine for cases 7 & 9 who are compound heterozygous for delEx2 and c.916T>C in DBT.
B) A significant reduction in the isoleucine level was not appreciated status post thiamine for case 12 who is homozygous for c.1261G>T in DBT.

## Slide 6
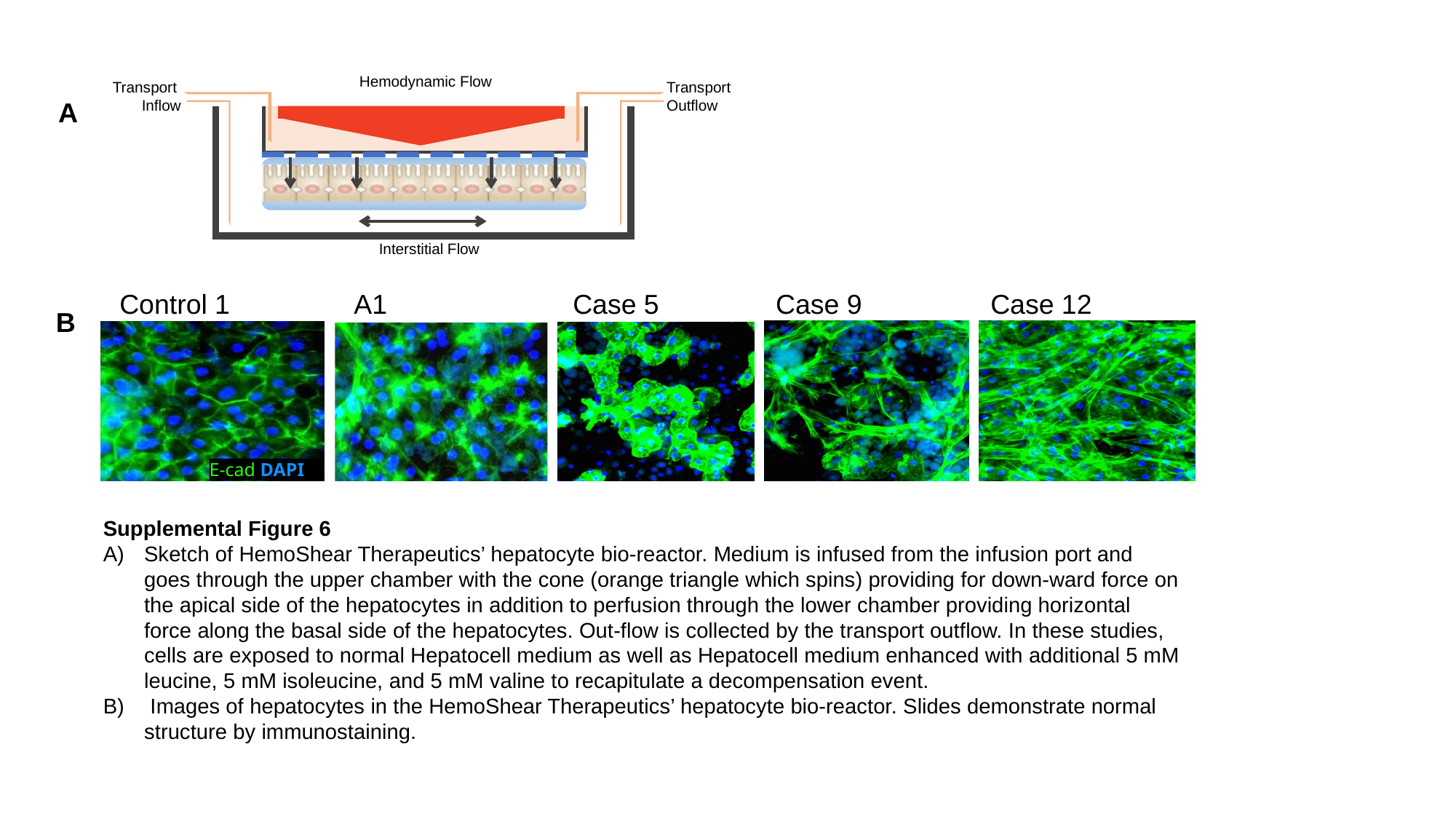

Hemodynamic Flow
Transport
Inflow
Transport
Outflow
Interstitial Flow
A
Control 1
A1
Case 5
Case 9
Case 12
E-cad DAPI
B
Supplemental Figure 6
Sketch of HemoShear Therapeutics’ hepatocyte bio-reactor. Medium is infused from the infusion port and goes through the upper chamber with the cone (orange triangle which spins) providing for down-ward force on the apical side of the hepatocytes in addition to perfusion through the lower chamber providing horizontal force along the basal side of the hepatocytes. Out-flow is collected by the transport outflow. In these studies, cells are exposed to normal Hepatocell medium as well as Hepatocell medium enhanced with additional 5 mM leucine, 5 mM isoleucine, and 5 mM valine to recapitulate a decompensation event.
 Images of hepatocytes in the HemoShear Therapeutics’ hepatocyte bio-reactor. Slides demonstrate normal structure by immunostaining.
