# Supplementary material for: Genomic and biochemical analysis of repeatedly observed variants in DBT in individuals with maple syrup urine disease of Central American ancestry
Source: Am J Med Genet A. 2022 Jul 7;188(9):2738–49. doi: 10.1002/ajmg.a.62893 (PMC9542135; doi:10.1002/ajmg.a.62893)
Supplement: Supplementary file 2 — Appendix S1 Supporting Information [file AJMG-188-2738-s001.docx]

**Supporting Information**

1. Materials and Methods
   1. Immunostaining

Hepatocytes plated on transwells and exposed to hemodynamics were fixed in 4% paraformaldehyde for 15 minutes, permeabilized in 0.2% Triton X-100 in PBS for 5 minutes and incubated with primary antibody for 2 hours at room temperature. After 3 washes in PBS, hepatocytes were incubated with secondary antibodies for 1 hour at room temperature. Anti-human primary antibodies against E-cadherin (Santa Cruz Biotechnology, sc-71009) were utilized, and goat anti-mouse conjugated to Alexa Fluor 488 (Life Technologies; A11001) were used as secondary antibodies. Nuclei were stained with DAPI (Thermo Fisher Scientific, D1306) or TO-PRO-3 (Life Technologies, T3605). Images of immunofluorescent-labeled cells were acquired with a Nikon 20× or 100× objective lens on a Nikon C1 confocal microscope with EZ-C1 v3.9 software or a Nikon 20× objective extra-long working distance lens on the ImageXpress Micro XLS Widefield High-Content Analysis System (Molecular Devices).

- 1. Branched-chain amino acid supplemented media preparation and exposure

To recreate a milieu representative of a patient with excess protein load, media supplemented with the addition of L-leucine, L-isoleucine, and L-valine (Cat# L8912, I7403, and V0513, Sigma Aldrich, St. Louis, MO) was prepared. The amino acids were prepared as a 20X or 100X stock and diluted to a final concentration of 5 mM. Cells were incubated in either regular media or amino acid supplemented media after 4 days of cell culture until the end of the experiment. A custom Modified Corning culture media for Hepatocells without branched-chain amino acids was formulated by adding low, moderate, and high levels of L-leucine, L-isoleucine, and L-valine to mimic different disease states.

1. Results
   1. Additional Case Summaries
      1. A1 is a Salvadoran female homozygous for NM_000709.4:c.288+1G>A [VCV000093351.15] in *BCKDHA* who presented on DOL 7 with mental status changes and a positive newborn screening consistent with MSUD. She initially had apnea and a movement disorder with leucine level of 2105 µM and required dialysis. She underwent liver transplantation at 2 years due to recurrent episodes of decompensation.
      2. A2 is a Salvadoran female compound heterozygous for NM_000709.4:c.288+1G>A;c.661_664del  [VCV000203638.3] in *BCKDHA* who presented on DOL 6 with poor oral intake and lethargy, and a positive newborn screening. Her initial leucine level was >2000 µM and she required dialysis. She had a liver transplantation at 3 years of age after multiple admissions for hyperleucinemia.
      3. A3 is a male homozygous for NM_000709.4:c.1312T>A(p.Tyr438Asn) [VCV000100009.20] in *BCKDHA* identified by newborn screening with an initial leucine level of 2265 µM and required dialysis. He had multiple episodes of decompensation and underwent liver transplant at 2.5 years.
      4. B1 is a male homozygous for deletion of exons 4-10 in *BCHDHB* who had a liver transplant at 19 months of age for improved stability.
      5. Case 12 is a Salvadoran male homozygous for NM_001918.5:c.1261G>T(p.Gly421Trp) [VCV000203669.2] in *DBT* who presented with lethargy, decreased oral intake, and a positive newborn screen on DOL 8. His initial leucine level was 2265 µM and he required dialysis. He underwent liver transplantation at 2.5 years after multiple episodes of decompensation.
2. Discussion
   1. Reclassification of NM_001918.5(DBT):c.1261G>T(p.Gly421Trp) [VCV000203669.2] from uncertain significance to likely pathogenic

The c.1261G>T variant was detected by next generation sequencing and confirmed by Sanger sequencing. The variant is classified as being of uncertain significance in ClinVar (Accession # VCV000203669.2). It is absent from gnomAD, has a CADD score (Hg38 v1.6) of 26.1, and a majority of computational predictors suggest it is pathogenic. A thiamine supplementation with isoleucine challenge was performed on Case 12 and this individual was determined to be nonresponsive to thiamine (Supplemental Figure 5B). Explanted hepatocytes from Case 12 showed neither *DBT* expression nor activity; there was no enzyme detected by Western Blot and a BCAA challenge demonstrated a 15-22-fold increase of branched-chain alpha-keto acid levels. Although it is unclear why this missense variant results in the absence of protein, it may impact either mRNA or protein stability by an unclear mechanism. Given the additional information obtained about this variant from this study, this variant should be classified as likely pathogenic.

**Supplemental Figures**

**Supplemental Figure 1**

Exon targeted array data indicating reduced hybridization for exon 2 probes. Control sample shows Exon 2 probes clustering around Log2 ratio of 0 indicating normal copy number. Case 10 and case 1 show progressively less exon 2 probe binding indicative of heterozygous and homozygous deletion of exon 2, respectively.

**Supplemental Figure 2**

**Sanger sequencing confirmation of *DBT* delEx2 variant detected by next generation sequencing**

1. Sanger sequencing confirms the deletion of exon 2 in Case 7. A 7bp area of homology with intron 1 and intron 2 is detected on either side of the roughly 7.4kb deletion.
2. Schematic of primer design utilized for confirmation of exon 2 deletion with primer sequences. For sequencing, PCR 3 has M13F and M13R tails; M13 sequences not shown.

**Supplemental Figure 3**

Parental testing to determine phase indicates c.916T>C in trans with deletion of exon 2 in Case 10. Sanger sequencing of Case 10 and his mother and exon targeted array for Case 10 and his father are shown indicating inheritance of each variant from a separate parent. For Sanger sequencing, the following primers were used:

Forward = CATATTGCATGTTGGCAAAGCAA

Reverse = CAGCCCTGAAGATACCTCATTTTGG

**Supplemental Figure 4**

**Possible area of identity by descent**

A) Chromosomal microarray analysis of Case 5 indicates a region of homozygosity surrounding the *DBT* locus. Depicted is a B allele Frequency plot of SNP microarray data. There is a ~15 Mb area of homozygosity surrounding *DBT* suggestive of a diplotype block with identity by descent between both copies of this area of chromosome 1.

B) A genotype concordance plot from residual exome data from Case 7 and Case 8, who are compound heterozygous for delEx2 and c.916T>C in *DBT*. Calls as reference or variant by position were graphed to show concordant and discordant genotypes between the two cases. There is a ~15Mb region (green shading) around *DBT* (dashed line) where there are no apparent discrepancies between the calls, suggesting an inherited diplotype block for these two cases who are not known to be related.

**Supplemental Figure 5**

Branched-chain amino acid analysis via dried blood spot at time points 0, 30, 90, 180, and 300 minutes following a 70 mg/kg isoleucine bolus at baseline and repeated after >1 week of thiamine supplementation (B1, 100mg).

A) A significant reduction in the isoleucine level was not appreciated status post thiamine for cases 7 & 9 who are compound heterozygous for delEx2 and c.916T>C in *DBT*.

B) A significant reduction in the isoleucine level was not appreciated status post thiamine for case 12 who is homozygous for c.1261G>T in *DBT*.

**Supplemental Figure 6**

A) Sketch of HemoShear Therapeutics’ hepatocyte bio-reactor. Medium is infused from the infusion port and goes through the upper chamber with the cone (orange triangle which spins) providing for down-ward force on the apical side of the hepatocytes in addition to perfusion through the lower chamber providing horizontal force along the basal side of the hepatocytes. Out-flow is collected by the transport outflow. In these studies, cells are exposed to normal Hepatocell medium as well as Hepatocell medium enhanced with additional 5 mM leucine, 5 mM isoleucine, and 5 mM valine to recapitulate a decompensation event.

B) Images of hepatocytes in the HemoShear Therapeutics’ hepatocyte bio-reactor. Slides demonstrate normal structure by immunostaining.
